# Evaluating Digital Maturity and Patient Acceptability of Real-Time Patient Experience Feedback Systems: Systematic Review

**DOI:** 10.2196/jmir.9076

**Published:** 2019-01-14

**Authors:** Mustafa Khanbhai, Kelsey Flott, Ara Darzi, Erik Mayer

**Affiliations:** 1 Centre for Health Policy Imperial College London London United Kingdom

**Keywords:** digital maturity, digital technology, feedback, patient experience, real time

## Abstract

**Background:**

One of the essential elements of a strategic approach to improving patients’ experience is to measure and report on patients’ experiences in real time. Real-time feedback (RTF) is increasingly being collected using digital technology; however, there are several factors that may influence the success of the digital system.

**Objective:**

The aim of this review was to evaluate the digital maturity and patient acceptability of real-time patient experience feedback systems.

**Methods:**

We systematically searched the following databases to identify papers that used digital systems to collect RTF: The Cochrane Library, Global Health, Health Management Information Consortium, Medical Literature Analysis and Retrieval System Online, EMBASE, PsycINFO, Web of Science, and CINAHL. In addition, Google Scholar and gray literature were utilized. Studies were assessed on their digital maturity using a Digital Maturity Framework on the basis of the following 4 domains: capacity/resource, usage, interoperability, and impact. A total score of 4 indicated the highest level of digital maturity.

**Results:**

RTF was collected primarily using touchscreens, tablets, and Web-based platforms. Implementation of digital systems showed acceptable response rates and generally positive views from patients and staff. Patient demographics according to RTF responses varied. An overrepresentation existed in females with a white predominance and in patients aged ≥65 years. Of 13 eligible studies, none had digital systems that were deemed to be of the highest level of maturity. Three studies received a score of 3, 2, and 1, respectively. Four studies scored 0 points. While 7 studies demonstrated capacity/resource, 8 demonstrated impact. None of the studies demonstrated interoperability in their digital systems.

**Conclusions:**

Patients and staff alike are willing to engage in RTF delivered using digital technology, thereby disrupting previous paper-based feedback. However, a lack of emphasis on digital maturity may lead to ineffective RTF, thwarting improvement efforts. Therefore, given the potential benefits of RTF, health care services should ensure that their digital systems deliver across the digital maturity continuum.

## Introduction

### Background of Patient Experience Feedback

Alongside measures of clinical effectiveness and safety outcomes, patient experience is increasingly recognized as an important indicator of the quality of health care provision and is frequently cited in health policies [1-3]. Yet, in practice and research, the concept of patient experience has had varied uses and is often discussed with little more explanation than the term itself [4]. In 2011, the English National Health Service (NHS) [5] outlined 8 domains that help define patient experience and are critical to patients’ experience of health care services. This has been used as an agreed working definition of patient experience to guide the measurement of patient experience across the NHS.

### Patient Experience Feedback in Real Time

One of the essential elements of a strategic approach to improving patients’ experience is to measure and report on patients’ experiences to assess progress, strengthen accountability, and identify new opportunities for improving performance [6]. Evidence suggests that this can be achieved using real-time feedback (RTF) [7]. RTF involves the systematic collection, analysis, and reporting of information from individuals at the point of care [8]. Previous studies have found that RTF has the potential to enable health care organizations to respond promptly to patients’ concerns and make timely improvements to services [7,9-11].

### Collecting Real-Time Feedback Using Digital Technology

With the ability to maximize the scalability and speed of data collection while reducing cost [12], digital tools (tablets, kiosks, emailed survey, and websites) are increasingly being utilized to gather patient experience feedback [13,14], allowing summary results to be reported on an ongoing [15] real-time basis. In a recent Cochrane review [16], self-administered survey questionnaire responses collected using mobile apps were compared with those collected using other methods, and it was concluded that the delivery of survey questionnaires through apps does not affect data equivalence and can improve data completeness. However, none of the questionnaires evaluated patient experience.

There is a growing pressure on health care services to embrace digital technologies tosignificantly improve the patient experience [17]. With an increase in adoption of digital systems pertaining to RTF, health care services must recognize and overcome the barriers that may hinder successful integration and uptake of such technologies. One of the ways of achieving this is through systematic evaluation and monitoring of digital systems to ensure they operate in the way they are intended and cultivate a better patient experience [17]. Digital maturity—the extent to which digital technologies are used as enablers to deliver a high-quality health service—is an emerging concept across developed health care systems [17]. Evidence suggests that digital maturity is linked to better outcomes and is indicative of a well-performing organization [18]. Evaluating digital systems for digital maturity highlights where gaps exist in maturity, which presents an opportunity to address the specific shortcomings [17]. The digital maturity of the systems that are being used to collect RTF has not been previously evaluated. A deeper understanding of the limitations of individual digital systems may help health care services pinpoint areas in need of improvement and organizational change. Without this, digital systems used for RTF may not be successful, and patients’ voices may go unheard.

Therefore, this systematic review aims to evaluate the digital maturity of digital RTF systems. Specific objectives were to (1) describe the digital modes utilized; (2) provide insights into patients’ and staff views of these digital systems; and (3) demonstrate digital maturity by systematically scoring individual digital systems.

## Methods

### Search Strategy

The following databases were searched: Medical Literature Analysis and Retrieval System Online, EMBASE, PsycINFO, The Cochrane Library (Cochrane Database of Systematic Reviews, Cochrane Central Register of Controlled Trials, Cochrane Methodology Register), Global Health, Health Management Information Consortium, CINAHL, and Web of Science. In addition, gray literature and Google Scholar were utilized to extract papers that were not retrieved in the databases searched. Publications from January 2000 to February 2017 were included. We limited our electronic searches to studies on or after 2000 because it was around this time when the digital technology revolution in health care began to emerge [19]. Owing to the diversity of terms used inferring patient experience, combinations of search terms were used. Multimedia Appendix 1 provides the complete list of subheadings (Medical Subject Headings) and keywords.

### Inclusion Criteria

Studies were deemed eligible for inclusion if (1) digital modes of administration were employed; (2) collection was in real time (at the point of care) or near real time (immediately after discharge); and (3) they were conducted in a primary or secondary care setting. There was no restriction on the age of the patient population of interest. Both quantitative and qualitative studies were included. Multimedia Appendix 1 provides further details of the inclusion criteria.

### Search Flow

The research adhered to the guideline presented in the Preferred Reporting Items for Systematic Reviews and Meta-Analyses (PRISMA) 2009 checklist [20]. Data analysis involved the comparison of included studies and extracted data. Due to the heterogeneous nature of the studies, a narrative synthesis was deemed most appropriate. This was followed by scoring the digital systems reported in the studies to determine the digital maturity.

### Digital Maturity

An existing framework [17] was utilized to determine the digital maturity of individual digital systems. This framework is embedded in the literature and has the benefit of systematically assessing the effectiveness of any digital system in health care. Each included study was scored across four key domains—capacity or resource, usage, interoperability, and impact. The framework highlights key questions for each domain and is based on “yes” or “no” response to each question. We assigned a maximum of 1 point if the digital system in each study demonstrated appropriate evidence for that particular domain. The maximum overall points a study could achieve was 4. This indicates the overall success of the digital platform [17]. Two reviewers (MK and KF) independently scored each study; disagreements in scoring were resolved by discussion between the two reviewers. Interrater agreement *kappa* was calculated.

## Results

### Overall Description

**Figure 1:**
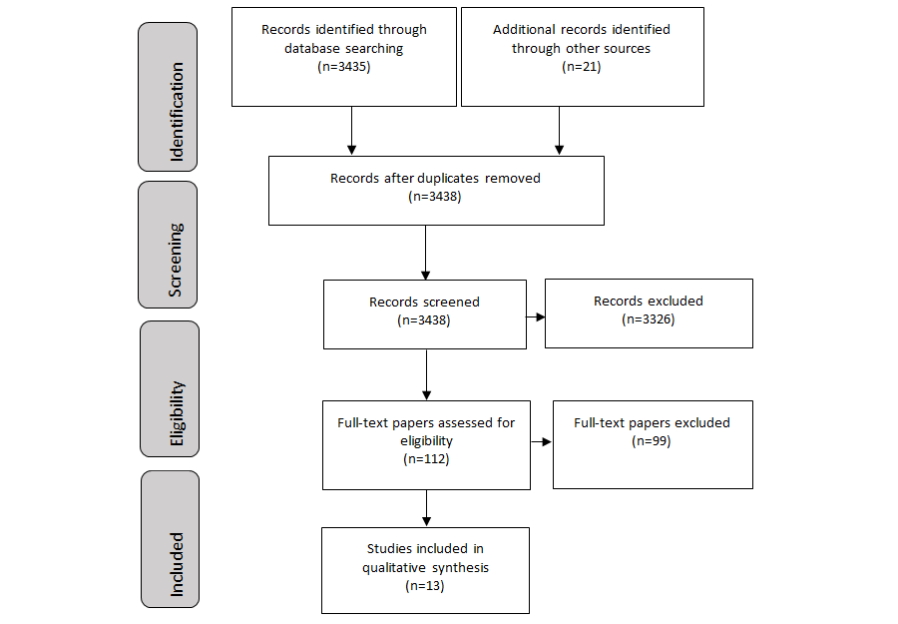
The Preferred Reporting Items for Systematic Reviews and Meta-Analyses 2009 checklist.

The initial search returned 3456 papers; after removing duplicates, 3438 papers were retained. The titles and abstracts were screened, and 112 papers were identified as potentially eligible for inclusion. Full-text papers were retrieved and assessed for inclusion, of which 13 were retained for final inclusion. RTF was defined as feedback collected while patients are in a hospital, at the point of care, while receiving care, or immediately after discharge. The main reason for exclusion was the papers did not report patient experiences or reported user experience of other digital technology. Figure 1 illustrates the PRISMA flowchart representing the study selection process and reasons for exclusion.

The reasons for exclusion of records (n=3326) and full-text papers (n=99) were the following: nondigital mode of administration (n=241), not real time or near real time (n=130), assessment or evaluation of other parameters (n=1489), feasibility or usability testing of digital systems not related to patient experience data collection (n=655), experience of other digital technology (n=848), and reviews (n=62).

### Systematic Review of Studies

#### Study Characteristics

Of the 13 studies included in the final review (Table 1), 5 were based in general practice [10,21-24] and the rest were based in an acute care hospital setting [9,25-31]. Of all the studies, 5 were from the United Kingdom [21,22,24,25,28], 7 from the United States [9,10,26,27,29-31], and 1 from Canada [23]. All but one [28] studies were based on adult populations. However, the experience was reported by adults (parents or relatives) for the study [28] conducted in the neonatal ward. Five studies were qualitative studies on patients’ or staff views [21,24] and on barriers to and facilitators [25,27,28] of RTF. The remainder of the studies used a quantitative approach with varied outcome measures, of which the recurring measures were response rates of RTF [9,10,22,23,27,29] and association with patient demographics, that is, age, gender, ethnicity, and literacy [9,22,23,31].

**Table 1:** Study characteristics of the 13 studies included in the systematic review.

Publication title; author(s), year	Study design	Duration of study	Types of survey questionnaire(s)	Mode of administration
Capturing patient experience: a qualitative study implementing real-time feedback in primary care; Carter M et al, 2016 [21]	Qualitative	3 months	Modified Staff opinions (semistructured interviews and focus groups) using Normalization Process Theory	Kiosks
Patients’ use and views of real-time feedback technology in general practice; Wright C et al, 2017	Exploratory randomized trial	3 months	Modified (amalgamated Friends and Family Test, 6 items focusing on access, communication and satisfaction [derived from general practitioner patient survey], 2 practices tailored questions^a^)	2 touch screens (1 Kiosk and 1 desktop device)
Measuring the patient experience in primary care. Comparing e-mail and waiting room survey delivery in a family health team; Slater M et al, 2016 [23]	Cross-sectional comparative analysis	1 month	Modified (amalgamated Commonwealth Fund International Health Policy Survey, patient demographics, self-rated health)	Tablet
Barriers and facilitators of a near real-time feedback approach for measuring patient experiences of hospital care; Kasbauer et al, 2017 [25]	Qualitative	10 months	Novel and validated (Compassionate Care Toolkit)	Tablet and kiosks
Real-time patient survey data during routine clinical activities for rapid-cycle quality improvement; Wofford et al, 2015 [10]	Feasibility study	1 month	Modified (dental care, waiting room experience, continuity, and internet access)	Tablet
Real-time patient experience surveys of hospitalized medical patients; Indovina et al, 2016 [26]	Prospective randomized	5 months	Previously validated (US Department of Health and Human Service and the Hospital Consumer Assessment of Healthcare Providers and Systems [HCAHPS])	Web-based platform
Evaluating patient-centred care (PCC): pilot study testing feasibility of electronic data collection in hospitalised older adults; Duffy et al, 2012 [27]	Cross-sectional feasibility	3 months	Previously validated (e-Caring Assessment Tool)	Tablet
Patient experience tracker (PET) survey as measure of quality in the neonatal unit; Aladangady et al, 2011 [28]	Qualitative	N/A^b^	Novel	Tablet
Development and validation of the tool to assess inpatient satisfaction with care from hospitalists; Torok et al, 2014 [29]	Cross-sectional	3 months	Novel and validated (Tool to Assess Inpatient Satisfaction with Care from Hospitalists)	Tablet Paper
Incentivized digital outcomes collection; Isenberg S et al, 2001 [30]	Feasibility study	3 months	Previously validated (Visit Rating Questionnaire)	Telephone (electronic voice response technology)
Exploring patients’ views toward giving Web-based feedback and rating to general practitioners in England: a qualitative descriptive study; Patel et al, 2016 [24]	Descriptive exploratory qualitative approach	N/A	Previously validated (Friends and Family Test)	Web-based platform (National Health Service Choices) Paper
Obtaining patient feedback at point of service using electronic kiosks; Dirocco et al, 2011 [9]	Feasibility study	1.5 months	Modified (National Committee for Quality Assurance’s Healthcare Effectiveness Data and Information Set and Quality Measurement standards)	Kiosk
Improving patient satisfaction through physician education, feedback, and incentives; Banka et al, 2015 [31]	Nonrandomized comparative study	14 months (7 months each year)	Previously validated (Assessing Residents’ Connect with patients, Introduce yourself and role, Communicate, Ask and anticipate, Respond, and Exit courteously and HCAHPS)	Paper and Web-based platform (results sent via email)

^a^Filter questions (tailored to patients visit).

^b^N/A: not applicable.

#### Modes of Feedback

RTF was collected using touchscreens (kiosk) [9,21,22,25], tablets [10,23,25,27-29], and Web-based platform [24,26,30,31] in the included studies. With regards to the timing of feedback, 11 studies collected their feedback in real time [9,10,21-23,25,27-31] and 2 studies in near real time [24,30] (ie, within 48 hours of discharge). Patient experience questionnaires used in each study varied; 5 studies modified an existing questionnaire [9,10,21-23], 5 used previously validated questionnaires [24,26,27,30,31], 2 used novel and validated questionnaires [25,29], and 1 used a novel and nonvalidated questionnaire [28]. The majority of the questionnaires were in English only.

#### Response Rates of Real-Time Feedback

Of the 13 studies, 6 studies [9,10,22,23,27,29] evaluated response rates (percentage) as part of their outcome measures. The response rates were 55.9% [23], 43.4% [10], and 2.5% [22] for studies conducted in primary care and 54.9% [9], 59.2% [27], and 61.5% [29] for those conducted in secondary care. Multimedia Appendix 2 demonstrates the absolute numbers of responses in each study identified above. Only 1 study evaluated response rates of RTF compared with non-RTF and showed that RTF improved response rates (55.9% vs 19.8%) [23].

#### Completion Time

Of all studies, 4 evaluated time to completion, and this varied from 40.4 seconds [10], <2 minutes [22], 3 minutes [9] to 31 minutes [27]. However, each questionnaire was different as it varied in length and number of questions.

#### Patient Demographics According to Real-Time Feedback Responses

The patient demographics that were collected varied, and 7 [9,22,23,25,27,29,31] of the 13 studies evaluated responses by patient demographics. Only Slater et al [23] compared RTF and non-RTF responses by patient demographics; RTF showed a higher percentage response in males (43.5 vs 36.6), in age (years) bracket 18-24 and 24-34 (6.7 vs 3.6 and 23.8 vs 18.5, respectively), and in those with lower literacy (34.4 vs 21.3) compared with non-RTF. Banka et al [31] revealed a higher percentage of male respondents (55.3 vs 41.4) with a white predominance (62.5 vs 60.9) using RTF compared with non-RTF. However, Wright et al [22] revealed different findings. There was a higher percentage response in females in the 46-65 (26.2%) and >65 (33.8%) age range and higher response from white than from ethnic patients. Dirocco et al [9] showed mixed results in that although the response was higher from females, there was a higher percentage response from African American individuals than from white American individuals (48.5 vs 38.6) and those aged 18-49 years (45.2). Torok et al [29] and Duffy et al [27] showed that the response rates from elderly patients were adequate; however, of these elderly patients, there was an overrepresentation in females [27,29], white individuals [27,29], and educated patients [27] who were aged ≥60 years. Kasbauer et al [25] showed that elderly patients responded to the survey but with the help of volunteers. Multimedia Appendix 3 summarizes the findings from the included studies.

#### Patient Views of Real-Time Feedback

Patients’ views of RTF were collected in 6 studies [9,10,22,24,27,30]. The main pros of RTF were as follows: easy to complete [10,22], easy to use [9,27], and willingness to use [24,27] the data collection tool. Isenberg et al [30] showed that patients were motivated to use RTF; however, this was incentivized with the reward of free long-distance minutes for patients and with a practice improvement program for the staff. Patel et al [24] identified that younger patients (aged <50 years) found digital platforms more accessible compared with older ones (aged ≥60 years). However, Duffy et al [27] further evaluated opinions of older adults and found a preference toward digital platforms; for example, 70% of patients preferred to answer questions using an iPad. Four studies found that patients thought RTF completion was quick with fast turnaround time [9,10,22,27]. The main cons of RTF were as follows: lack of awareness of the opportunity to leave feedback [22,24], lack of time [22], concerns over technology [22,24], concerns over anonymity [22,24], and age- or disease-related exclusion [24,27]. Interestingly, Wright et al [22] showed that those who did not use RTF were still positive about the idea of providing RTF.

#### Staff Views of Real-Time Feedback

Staff views were collected in 4 studies [9,21,25,28]. Positive staff views toward RTF were as follows: immediacy of RTF compared with traditional surveys, which helped offset “feedback fatigue,” complemented other forms of feedback with the potential of integrating with other data sources [21]. Staff found the RTF data was felt to be useful when summarized, highlighting areas of improvement at-a-glance on a dashboard [25], and when there was coworking with senior clinical staff [25], increasing staff morale and awareness of RTF. In some studies, free text was found to be more useful than quantitative questions [21,25] as it brought the experiences to life for frontline staff and added a “sense of urgency” to address them in improvement efforts [25]. Concerns included duplication with other forms of feedback, lack of time for patients to reflect on experience, extreme views, exclusion of certain patient groups, staff did not feel included in decision making [21], initial reluctance [28], limited time to review, and lack of access to the results [25].

#### Evaluating Digital Maturity of Real-Time Feedback Systems

Three studies received a score of 3 [10,25,30], 2 [9,21,22], or 1 [27,28,31] points, respectively, while 4 studies [23,24,26,29] were attributed 0 points. While 7 studies demonstrated capacity or resource, 8 demonstrated impact. None of the studies demonstrated digital systems that were deemed to be mature, that is, did not achieve a full score in all of the 4 domains. We describe in detail how the digital system in each study demonstrated evidence that determined whether a point was given in each of the 4 domains. Following independent scoring by MK and KF, Cohen *kappa* was calculated as 0.98, suggesting an almost perfect agreement. There was only one domain where the scoring differed (Usage) [26], and through discussion, a final score of 0 was inputted. Multimedia Appendix 4 details the individual scoring with a description on each domain.

## Discussion

### Principal Findings

This review highlights that digital modes of administration of RTF are well accepted by patients and staff, with response rates equivalent to, and in some studies better than, non-RTF. From a patient’s perspective, it has been reported that its influx alongside demographic shifts such as increasing aging population and ethnicity [32,33] can lead to patient disengagement and poor uptake of these technologies [34]. On the contrary, we have shown that patients are in fact willing to engage with this technology for experience reporting, thereby disrupting previous nonreal-time, paper-based feedback. However, digital technology may not be preferred by all patients; therefore, health care organizations need to be mindful and consider other means of inclusivity when developing digital health care systems. From a staff perspective, although RTF is well received, problems arising from the lack of robust digital infrastructure [25] can thwart improvement efforts. From a health care organizational perspective, most digital systems were unable to demonstrate interoperability and very few demonstrated impact and, therefore, were not deemed digitally mature, compromising success within the organization.

### Digital Maturity of Existing Real-Time Feedback

By nature, maturity frameworks not only identify components of a successful system but also capture the evolution of a digital system from conception to implementation to impact. Using evidence where possible from the included studies, we highlight how each of the 4 domains contribute to ensuring digital maturity.

### Capacity or Resource

The success of digital health is contingent on establishing the necessary capacity and resources to build, use, and support access to high-quality health services and harvest useful information in the health system. There was a general lack of analytical support in most digital systems to extract valuable information such as the ability to examine user-specific interaction [10] or adjust survey responses [23]. A Kings’ Fund report explains that gleaning information from experience data requires the same analytical capability as interpreting clinical data; however, this is often unavailable [35]. The resource capacity requirements stretch beyond health professionals and technology specialists across the continuum of care to include health information managers to information security professionals [36]. Staff time was an important barrier in data collection, and this is in keeping with other studies that reported a lack of time or resources to collect, analyze, or act on data and need for staff training in data analysis and statistics to facilitate full understanding and use of results [37-39]. To circumvent this, some of the studies used volunteers [25,31] and incentives [30,31] to gather data. This generates concerns such as response bias and competition among clinicians, and there may be an element of the Hawthorne effect, whereby clinicians modify their behavior as they are being observed, which may explain the positive outcomes in those studies. Going forward, a strategic approach should take into consideration building human and institutional capacity, nurturing clinical and community champions, and developing the base of knowledgeable users to drive appropriate adoption of digital systems [36].

### Usage

As the needs and experiences differ greatly across these groups, a flexible or responsive data collection mode is needed, which can aid patients during the data collection process. While most patients in the included studies were comfortable using the digital system and required little prompting or help once they engaged with the survey, some differences were noted. Concerns exist that older patients are less comfortable with technology [40]; however, there was an overrepresentation in responses from patients aged >65 years in the included studies [22,25,27,29,31]. Moreover, some of these studies were conducted in the elderly population [25,27]. Despite this, the response rate in this patient group was adequate, suggesting that their movement into digital life is evolving. Furthermore, when certain conditions are met such as providing a supportive, nonhurried environment [41]; bold, plain, and large font with fewer graphics; and avoidance of certain colors [42], older adults can be successful users. The use of videos in the software can enhance accuracy and acceptability [10]. Furthermore, trained volunteers can provide a responsive approach to real-time data collection from lesser heard groups [14], increasing patient engagement and, subsequently, improving response rates. In addition, they can help reduce the data collection burden, which may otherwise fall on clinical staff and may even account for the false positives as seen in some surveys [43] due to the presence of staff during survey completion.

If data collection is obtrusive, unrealistic, or inaccurate, it can undermine enthusiasm for assessing and improving practice quality [44,45]. However, the digital systems in this review demonstrated quick turnaround of data collection, collation, and dissemination of results. This was key to successful implementation of digital systems as it promoted “buy-in” from staff. Furthermore, patients are fatigued of requests for feedback in health care and in daily life; hence, data collection should be quick, focused, and part of routine care to encourage participation from patients and clinicians and to be sustainable in a busy setting [10].

### Interoperability

From a health care perspective, interoperability is needed to reform the chaotic, and, at times, dysfunctional nature of how information is shared within and among health care services. There was a ubiquitous shortfall in achieving interoperability among the digital systems in this review. Data that are not interoperable cannot be analyzed alongside other data indicative of care quality. This perpetuates the siloed approach to data interpretation and creates a chasm between data and information for improvement. Some of the challenges in achieving interoperability include resistance from some vendors, prohibitively high data exchange fees, and lack of incentives to develop interoperability and technical variations. Without tackling the interoperability of RTF systems, it is likely that health care organizations will fail on delivering impactful quality improvement. This must now be a major focus for health care services, but it should be done in an organized way that prioritizes interoperability so that patient feedback data can communicate seamlessly to generate information that will benefit health care services and patients alike.

### Impact

To genuinely demonstrate impact, the digital system should not only be able to generate quality improvement activities but also demonstrate sustainability and cost-effectiveness. Three studies [10,25,30] achieved a score for impact as they demonstrated a change in practice following the implementation of RTF. However, the majority of the digital systems were not able to demonstrate any impact. The duration of studies in this review was short (between 1 and 12 months), and the sustainability of the digital system to continue to deliver quality improvement could not be evaluated. Without considerations of sustainability, digital programs are of limited value.

### Limitations

Due to publication bias, there may be unpublished evidence of nonsignificant or negative findings or findings held locally, which are not published or otherwise publicly available. We ascribed a level of digital maturity of individual systems based on the evidence provided in the studies. If this was not discernible while reviewing the studies, we assumed it to be lacking. As the remit of the included papers differed greatly, their digital systems may, in fact, have existing procedures for ensuring capacity, usage, interoperability, and impact, but the authors did not specify this information in the published paper. Of note, the studies were not evaluated for research quality before being included in the synthesis owing to the limited number of studies that were identified after exclusion.

### Conclusions

There was not a large body of published literature on digital modes in relation to RTF. However, the evidence provided by the studies in this review demonstrates that there is a potential in using digital modes of administration of RTF as an agent for improving service delivery. Patients and staff alike are willing to engage in RTF, demonstrated by acceptable response rates. However, for RTF to be impactful, health care organizations must ensure that they have strategies in place to deliver across all levels of digital maturity. Health care services have the capacity to introduce digital solutions for RTF; however, lack of interoperability is slowing down progress. In addition, it is possible that some health care services may be wasting effort and resources when they invest in digital technologies for RTF. On balance, the direction of the health care ecosystem toward embracing digital technology looks promising, and as health care shifts toward a patient-centered model, digital technology will be an important partner in this transformation.

## Appendix

Multimedia Appendix 1 Subheadings (MESH) and Keywords.

Multimedia Appendix 2 This demonstrates the percentage response rates for RTF completion including absolute number of patients in each study.

Multimedia Appendix 3 Response rate in percentage and representation of responses according to patients’ demographics documented from the studies in the systematic review.

Multimedia Appendix 4 Individual scores in relation to the digital maturity framework domains. A score of one indicates that the study demonstrates evidence in that particular domain, otherwise a score of zero is ascribed.

## Abbreviations

NHS: National Health Service
NIHR: National Institute for Health Research
PRISMA: Preferred Reporting Items for Systematic Reviews and Meta-Analyses
RTF: real-time feedback
