# Supplementary material for: Evaluating Digital Maturity and Patient Acceptability of Real-Time Patient Experience Feedback Systems: Systematic Review
Source: J Med Internet Res. 2019 Jan 14;21(1):e9076. doi: 10.2196/jmir.9076 (PMC6682271; doi:10.2196/jmir.9076)
Supplement: Multimedia Appendix 1 [file jmir_v21i1e9076_app1.pdf]

Multimedia Appendix 1

Subheadings (MESH) and Keywords

“Feedback” OR “Surveys and Questionnaires” OR “Data Collection” OR “Health Care Surveys” OR “Assessment” OR “Evaluation” AND “Patient-Centred Care” OR “Patient Satisfaction” OR “Patient Experience.mp” OR “Patient Experience.tw” OR “Physician-Patient Relations” AND “Computers, Handheld”, OR “Mobile Applications” OR “Cell phones” OR “User-Computer Interface” OR “Medical Informatics Applications” OR “Smartphone” OR “Digital” “Real-time.tw” OR “real-time.mp”

Inclusion Criteria

Timing of Feedback

Only data collected in *real time*, or *near real time*. This will include feedback that will be conducted while patients are in a hospital, at the point of care, while receiving care, or immediately after discharge.

Types of Technology

All mobile electronic devices, mobile phones; personal digital assistants (PDA) and PDA phones (eg, Blackberry, Palm Pilot), Smartphones (eg, iPhone), handheld and ultraportable computers such as tablet PCs (eg, iPad) and Smartbooks. Touchscreen devices (eg, Kiosks), bedside terminals, will be included. We will include personal desktop computers, notebook (laptop) computers if the data are inputted and collected in real time; this will, therefore, include Web-based patient experience questionnaires that have not been formatted to be compatible with the mobile operating system (OS).

Types of Participants

There will be no limits on study participants in terms of age, gender, ethnicity, morbidities (for patients, carers, or relatives) or staff role and occupation (for health care professionals, eg, nurse, surgeon, or physiotherapist). There will be no limits on the study setting, and we will include studies at all levels of health care setting (ie, primary, secondary and tertiary health care) and those conducted in the community.

Types of Outcome Measures

The review will consider studies that include the following: (1) questionnaire/survey design (ie, was a questionnaire designed for that specific technology and was there coproduction during the design process, statistical validity, and cognitive testing), language and understandability, data accuracy, data completeness; (2) patient factors, that is, functionality, human-computer interaction, response rates, personal, proxy and carer responses (for patients not capable of completing any surveys), patient demographics, disease groups, and different care episodes; (3) staff and organizational factors, staff time, response to cultural shift, hospital profitability and cost-effectiveness. All studies must include patients experience as per the NHS Patient Experience Framework, which outlines 8 domains defined as critical to

a “good” patient experience, including respect, information and communication, physical comfort, emotional support, and access to care.

Types of Studies

The review will include randomized controlled trials, nonrandomized controlled trials, case-control studies, prospective and retrospective cohort studies, and qualitative studies. We will consider all studies that incorporate patient experience using real-time tools. We will exclude studies that gather PROMs, symptom monitoring, symptom information, quality of life measures, and ecological momentary assessment without patient experience data.
