# Supplementary material for: Evaluating Digital Maturity and Patient Acceptability of Real-Time Patient Experience Feedback Systems: Systematic Review
Source: J Med Internet Res. 2019 Jan 14;21(1):e9076. doi: 10.2196/jmir.9076 (PMC6682271; doi:10.2196/jmir.9076)
Supplement: Multimedia Appendix 2 [file jmir_v21i1e9076_app2.pdf]

Multimedia Appendix 2

	Number of patients who use RTF/total number of patients	Percentage response rate
Slater et al	606/1084	55.9%
Wofford et al	316/727	43.4%
Wright et al	1941/79145	2.5%
Dirocco et al	1766/3219	54.9%
Duffy et al	90/152	59.2%
Torok et al	203/330	61.5%
