# Supplementary material for: Evaluating Digital Maturity and Patient Acceptability of Real-Time Patient Experience Feedback Systems: Systematic Review
Source: J Med Internet Res. 2019 Jan 14;21(1):e9076. doi: 10.2196/jmir.9076 (PMC6682271; doi:10.2196/jmir.9076)
Supplement: Multimedia Appendix 3 [file jmir_v21i1e9076_app3.pdf]

		Slater et al	Wright et al	Dirocco et al	Duffy et al	Torok et al	Kasbauer et al	Banka et al	Wofford et al
Overall response rate		55.9%	3.2%	55%	59%	61.5%	N/A	N/A	43.4%
Age	*18-49*	✓		✓					
	50-65		✓					✓	
	>65		✓		✓	✓	✓	✓	
Sex	*M*	✓						✓	
	F		✓	✓	✓	✓			
Ethnicity	*White*		✓		✓	✓		✓	
	Other			✓					
Literacy	*Low*	✓							
	High				✓				

Multimedia Appendix 3. Response rate in percentage and representation of responses according to patients' demographics documented from the studies in the systematic review.

NOTE: A tick indicates an overrepresentation in that particular patient group. Some studies did not report or evaluate response rates according to demographic profiles.
