# Supplementary material for: Evaluating Digital Maturity and Patient Acceptability of Real-Time Patient Experience Feedback Systems: Systematic Review
Source: J Med Internet Res. 2019 Jan 14;21(1):e9076. doi: 10.2196/jmir.9076 (PMC6682271; doi:10.2196/jmir.9076)
Supplement: Multimedia Appendix 4 [file jmir_v21i1e9076_app4.pdf]

Multimedia Appendix 4. Individual scores in relation to the digital maturity framework domains. A score of one indicates that the study demonstrates evidence in that particular domain, otherwise a score of zero is ascribed.

Digital Maturity Framework				
	**Capacity/resource**<br>*The resources available for a system, including the organizational readiness and individual abilities needed to use a digital system correctly*	**Usage**<br>*The actual uptake of a system, or the degree to which it is used by a range of people who need to input it or otherwise access it*	**Interoperability**<br>*The capability it has to communicate across services or other operating Information Technology systems*	**Impact**<br>*The impact it has in terms of both outcomes for patients and structure, process, and finances*
**Kasbauer et al**	**1**<br>Ongoing support ensuring sufficient resource, capacity, and flexibility. Volunteers engaged with data collection, freeing up staff to access reports. However, there was little to no access to computers for staff to review the reports and limited time for staff to access the reports.	**1**<br>Main driver for successful action planning and implementation of improvement was having senior clinical staff working alongside frontline staff. However, there were initial reports of tablets shutting down during a scheduled update, and tablets did not transmit survey responses owing to the loss of 3G or wireless connectivity.	**0**<br>No evidence on the interoperability was demonstrated.	**1**<br>The digital approach benefited from various support structures and systems to minimize complications or burden placed on both staff and volunteers.
**Wofford et al**	**1**<br>Department had a long history of tablet computers in patient care activities, good wireless system, and faculty and staff with active interest in computer-assisted patient education and quality improvement.	**1**<br>The software performed well regarding the survey creation and deployment of tablet computers, cloud capture of response, and accumulation and display of data. However, it did not allow the use of graphics/video, which would enhance the accuracy and acceptability. The average completion	**0**<br>No evidence on the interoperability was demonstrated.	**1**<br>The digital platform resulted in establishing and increasing access to certain services (dental) as well as revision of outpatient policies (waiting room).

		time was 40.4 seconds.		
**Isenberg et al**	**1**<br>A good number of clinicians involved in the program with every individual being supplied with confidential user ID and password to review individual patient scores and the date of response.	**1**<br>There was an increase in participation by unifying common use of readily available telephone (via interactive voice response technology) and the internet. The digital program dichotomized results to allow for easier comparison of the results. The study design involved physician and patient incentive.	**0**<br>No evidence on the interoperability was demonstrated.	**1**<br>The digital program provided benchmarking and linked percentage excellent responses to employee compensation.
**Carter et al**	**1**<br>Information about the digital program was shared throughout the practice and with enthusiasm for new initiatives, RTF was received positively as the immediacy helped offset “feedback fatigue” and was incorporated into practice routine and process.	**1**<br>With good communication between staff groups (medical, nursing, administrative, and reception staff) and individuals, it fostered a sense of involvement, which achieved buy-in from staff. However, there was a lack of availability of kiosks that impacted the use.	**0**<br>No evidence on the interoperability was demonstrated.	**0**<br>Owing to short time frame (12 weeks), impact of the digital system was not assessed.
**Wright et al**	**1**<br>One member of staff had oversight of the digital program; however, instead of clinicians, receptionists were given responsibility to encourage patients to use the digital program.	**1**<br>Feedback reports were generated; team level reports were emailed to practice manager for dissemination to wider team and personalized reports emailed directly to individual clinician. Patients (80.6%) found the survey easy to complete (<2	**0**<br>No evidence on the interoperability was demonstrated.	**0**<br>Pilot study precluded long-term follow-up in respect of whether changes in service provision had been considered, introduced and sustained. There was an assessment of the cost of the digital system, but no evidence on cost-

		minutes).		effectiveness was documented.
**Dirocco et al**	**1**<br>An introduction of the digital system did not hinder office flow in terms of wait time to check in and check out noted from patients, providers, and staff. There was an external dedicated monitor to direct patient toward the kiosk at checkout.	**1**<br>Participants and patients were comfortable using the system and required little prompting with the minimal provider and staff effort to direct all patients to the kiosk. Time to completion was an average of 3 minutes per participant.	**0**<br>No evidence on the interoperability was demonstrated.	**0**<br>Showed capability of a digital platform to capture patients experience but did not assess resultant quality improvement.
**Aladangady et al**	**1**<br>Neonatal consultant, sister, and operational manager met with trust lead for the digital system and discussed with senior medical and nursing team, followed by further discussions with junior nursing and medical staff, ward clerks, health care assistance, housekeepers who unanimously agreed to adopt the digital system.	**0**<br>Poor placement resulted in a lack of use, data failing to reach the experience office, and device failure. Staff needed constant reminders to ensure use.	**0**<br>No evidence on the interoperability was demonstrated.	**0**<br>Despite efforts from the team, there was no demonstrable impact of the digital system.
**Duffy et al**	**0**<br>Digital system was used by a research assistant with little or no staff involvement.	**1**<br>The average time to complete the survey was 31 minutes, with 5 minutes to set up and log on to the device. It captured unit and patient characteristics, score individual items, gather feasibility and functionality data.	**0**<br>No evidence on the interoperability was demonstrated.	**0**<br>Demonstrated feasibility in older patients but no demonstrable impact of the digital system.
**Banka et al**	**0**<br>Digital system was terminated every July as volunteers were away on summer break.	**1**<br>Physicians used feedback scores, which was incentivized by monthly recognition of physicians who	**0**<br>No evidence on the interoperability was demonstrated.	**0**<br>No demonstrable impact from the digital system; however, the potential of cost-saving from using

		stood out on the survey.		volunteers, and system can minimize loss to hospital revenue but no evidence to support this claim.
**Indovina et al**	**0**<br>No evidence to support this.	**0**<br>Paper-based data collection, therefore, resource-intensive.	**0**<br>No evidence on the interoperability was demonstrated.	**0**<br>No demonstrable impact of the digital system.
**Slater et al**	**0**<br>No evidence to support this.	**0**<br>No evidence of usage.	**0**<br>No evidence on the interoperability was demonstrated.	**0**<br>No demonstrable impact of the digital system.
**Torok et al**	**0**<br>No evidence to support this.	**0**<br>No evidence of usage.	**0**<br>No evidence on the interoperability was demonstrated.	**0**<br>No demonstrable impact of the digital system.
**Patel et al**	**0**<br>No evidence to support this.	**0**<br>No evidence of usage.	**0**<br>No evidence on the interoperability was demonstrated.	**0**<br>No demonstrable impact of the digital system.
